# 24-Acetyl-8,11,14-trioxa-24,27-diaza­penta­cyclo­[19.5.1.1^22,26^.0^2,7^.0^15,20^]octa­cosa-2,4,6,15(20),16,18-hexaen-28-one

**DOI:** 10.1107/S1600536812027274

**Published:** 2012-06-23

**Authors:** Le Tuan Anh, Truong Hong Hieu, Anatoly T. Soldatenkov, Nadezhda M. Kolyadina, Victor N. Khrustalev

**Affiliations:** a Department of Chemistry, Vietnam National University, 144 Xuan Thuy, Cau Giay, Hanoi, Vietnam; b Organic Chemistry Department, Russian Peoples Friendship University, Miklukho-Maklaya St 6, Moscow, 117198, Russia; c X-Ray Structural Centre, A.N. Nesmeyanov Institute of Organoelement Compounds, Russian Academy of Sciences, 28 Vavilov St, B-334, Moscow 119991, Russian Federation

## Abstract

The title compound, C_25_H_28_N_2_O_5_, is a product of the Petrenko–Kritchenko condensation of *N*-acetyl­piperidone with 1,5-bis­(2-formyl­phen­oxy)-3-oxapentane and ammonium acetate. The mol­ecule comprises a fused penta­cyclic system containing an aza-14-crown-3-ether macrocycle, two piperidone and two benzene rings. The aza-14-crown-3-ether ring adopts a bowl conformation. The dihedral angle between the benzene rings fused to the aza-14-crown-4-ether unit is 70.18 (4)°. The central piperidone ring has a boat conformation, whereas the terminal piperidone ring adopts a chair conformation. The conformation of the central piperidone ring is determined by two intra­molecular N—H⋯O hydrogen bonds. In the crystal, mol­ecules are linked by weak C—H⋯O inter­actions into chains along [010].

## Related literature

For general background to the design, synthesis and applications of macrocyclic ligands for coordination and supra­molecular chemistry, see: Hiraoka (1978 ▶); Pedersen (1988 ▶); Gokel & Murillo (1996 ▶); Bradshaw & Izatt (1997 ▶). For related compounds, see: Levov *et al.* (2006 ▶, 2008 ▶); Komarova *et al.* (2008 ▶); Anh *et al.* (2008 ▶, 2012*a* ▶,*b* ▶); Hieu *et al.* (2011 ▶); Khieu *et al.* (2011 ▶); Sokol *et al.* (2011 ▶).
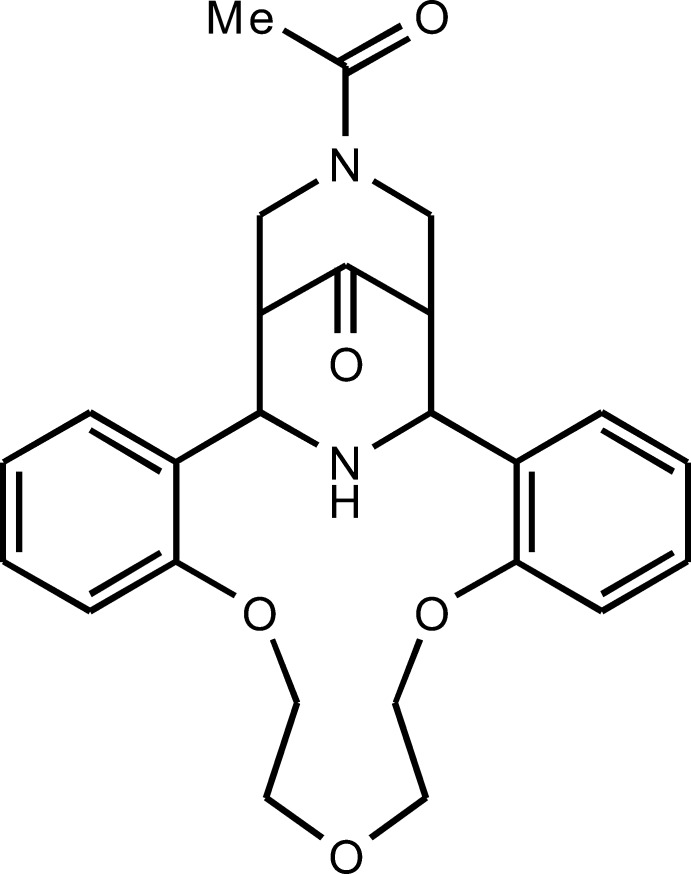


## Experimental

### 

#### Crystal data

C_25_H_28_N_2_O_5_
*M* *_r_* = 436.49
Orthorhombic, 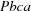

*a* = 17.1756 (6) Å
*b* = 11.1724 (4) Å
*c* = 22.6546 (8) Å
*V* = 4347.3 (3) Å^3^
*Z* = 8
Mo *K*α radiation
μ = 0.09 mm^−1^
*T* = 100 K
0.30 × 0.25 × 0.25 mm

#### Data collection

Bruker APEXII CCD diffractometer
Absorption correction: multi-scan (*SADABS*; Sheldrick, 2003 ▶) *T*_min_ = 0.973, *T*_max_ = 0.977
54466 measured reflections
6326 independent reflections
4682 reflections with *I* > 2σ(*I*)
*R*_int_ = 0.069

#### Refinement

*R*[*F* ^2^ > 2σ(*F* ^2^)] = 0.042
*wR*(*F* ^2^) = 0.106
*S* = 1.00
6326 reflections
293 parameters
H atoms treated by a mixture of independent and constrained refinement
Δρ_max_ = 0.34 e Å^−3^
Δρ_min_ = −0.24 e Å^−3^

### 

Data collection: *APEX2* (Bruker, 2005 ▶); cell refinement: *SAINT* (Bruker, 2001 ▶); data reduction: *SAINT*; program(s) used to solve structure: *SHELXTL* (Sheldrick, 2008 ▶); program(s) used to refine structure: *SHELXTL*; molecular graphics: *SHELXTL*; software used to prepare material for publication: *SHELXTL*.

## Supplementary Material

Crystal structure: contains datablock(s) global, I. DOI: 10.1107/S1600536812027274/aa2068sup1.cif

Structure factors: contains datablock(s) I. DOI: 10.1107/S1600536812027274/aa2068Isup2.hkl

Supplementary material file. DOI: 10.1107/S1600536812027274/aa2068Isup3.cml

Additional supplementary materials:  crystallographic information; 3D view; checkCIF report

## Figures and Tables

**Table 1:** Hydrogen-bond geometry (Å, °)

*D*—H⋯*A*	*D*—H	H⋯*A*	*D*⋯*A*	*D*—H⋯*A*
N27—H27⋯O8	0.90 (2)	2.49 (2)	3.0337 (13)	119 (1)
N27—H27⋯O14	0.90 (2)	2.44 (1)	3.0193 (13)	122 (1)
C21—H21⋯O28^i^	1.00	2.48	3.4683 (14)	168
C30—H30*B*⋯O28^i^	0.98	2.51	3.0556 (16)	115

Symmetry code: (i) 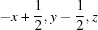.

**Fig. 1.**
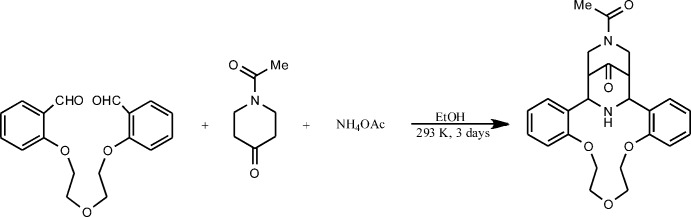
Petrenko-Kritchenko condensation of the N-acetylpiperidone with 1,5-bis(2-formylphenoxy)-3-oxapentane and ammonium acetate.

**Fig. 2.**
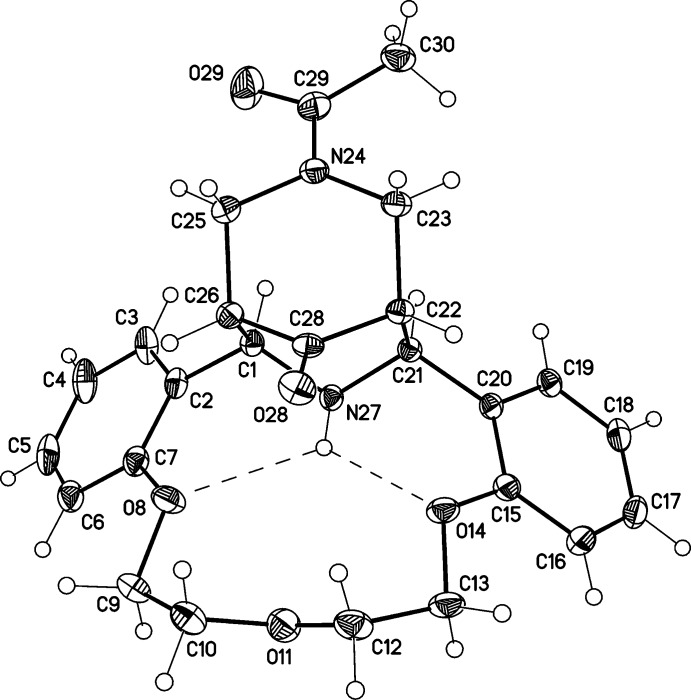
Molecular structure of I. Displacement ellipsoids are shown at the 50% probability level. H atoms are presented as small spheres of arbitrary radius. Dashed lines indicate the intramolecular N–H···O hydrogen bonds.

**Fig. 3.**
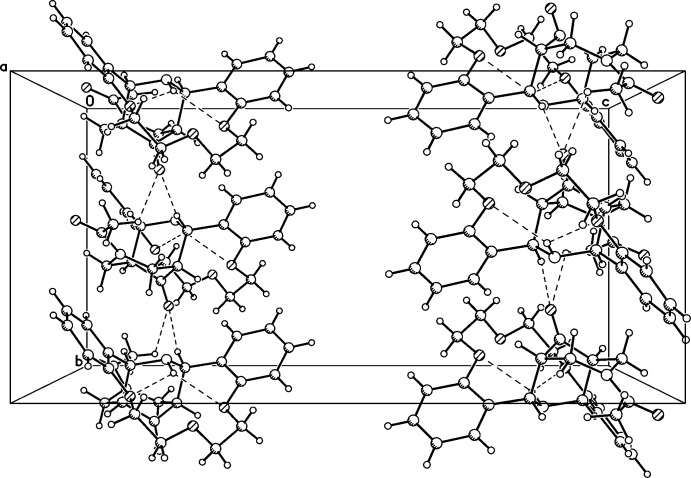
The H-bonded chains of I along the b axis. Dashed lines indicate the intramolecular N–H···O and intermolecular C–H···O hydrogen bonds.

## supplementary crystallographic information

### Comment

Design, synthesis and applications of macrocyclic ligands for coordination and supramolecular chemistry draw very great attention of investigators during the last several decades (Hiraoka, 1978; Pedersen, 1988; Gokel & Murillo, 1996; Bradshaw & Izatt, 1997). Recently we have developed the effective methods of synthesis of azacrown ethers containing piperidine (Levov *et al.*, 2006, 2008; Anh *et al.*, 2008, 2012*a*, 2012*b*), perhydropyrimidine (Hieu *et al.*, 2011), perhydrotriazine (Khieu *et al.*, 2011) and bispidine (Komarova *et al.*, 2008; Sokol *et al.*, 2011) subunits.

In attempts to apply this chemistry for obtaining of a macrocyclic ligand containing *N*-acylsubstituted bispidine moiety, we studied the Petrenko-Kritchenko condensation of the *N*-acetylpiperidone with 1,5-bis(2-formylphenoxy)-3-oxapentane and ammonium acetate. The reaction have proceeded smoothly to give the expected azacrown system with a good yield (Fig. 1).

The molecule of the title compound, C_25_H_28_ N_2_O_5_, comprises a fused pentacyclic system containing the aza-14-crown-3-ether macrocycle, two piperidone and two benzene rings (Fig. 2). The aza-14-crown-3-ether ring adopts a bowl conformation. The configuration of the C7—O8—C9—C10—O11—C12—C13—O14—C15 polyether chain is t–g(-)–t–t–g(+)–t (t = *trans*, 180°; g = *gauche*, ±60°). The dihedral angle between the planes of the benzene rings fused to the aza-14-crown-4-ether moiety is 70.18 (4)°. The central piperidone ring has a boat conformation, whereas the terminal piperidone ring adopts a chair conformation. Apparently, the conformation of the central piperidone ring is determined by the two intramolecular N–H···O hydrogen bonds (Table 1). The nitrogen N24 atom has a trigonal-planar geometry (sum of the bond angles is 359.8°), while the nitrogen N27 atom adopts a trigonal-pyramidal geometry (sum of the bond angles is 326.7°).

The molecule of the title compound possesses four asymmetric centers at the C1, C21, C22 and C26 carbon atoms and can have potentially numerous diastereomers. The crystal of the title compound is racemic and consists of enantiomeric pairs with the following relative configuration of the centers: *rac*-1*R**, 21*S**,22*R**,26*S**.

In the crystal, the molecules are bound by the weak intermolecular C–H···O hydrogen bonding interactions into the chains along [010] (Fig. 3, Table 1). The crystal packing of the chains is stacking along the *a* axis (Fig. 3).

### Experimental

Ammonium acetate (3.0 g, 39.0 mmol) was added to a solution of 1,5-bis(2-formylphenoxy)-3-oxapentane (3.14 g, 10.0 mmol) and *N*-acetylpiperidone (1.41 g, 10.0 mmol) in ethanol-acetic acid mixture (30 ml 1 ml). The reaction mixture was stirred at 293 K for 3 days (monitoring by TLC until disappearance of the starting heterocyclic ketone spot). At the end of the reaction, the formed precipitate was filtered off, washed with ethanol and re-crystallized from ethanol to give 2.54 g of white crystals of the title compound. Yield is 58%. *M*.p.= 500–502 K. IR (KBr), ν/cm^-1^: 1603, 1649, 1713, 3405, 3460. ^1^H NMR (CDCl_3_, 400 MHz, 300 K): δ = 2.37 (s, 3H, CH_3_C=O), 2.91 (m, 3H, H22, H26 and H27), 3.47 and 4.98 (both dd, 1H each, H1 and H21, *J =* 7.3 and 1.1), 3.92–4.10 (m, 12H, OCH_2_CH_2_OCH_2_CH_2_O, 2H23 and 2H25), 6.75–6.95 (m, 3H, H_arom_), 7.21–7.36 (m, 5H, H_arom_). Anal. Calcd. for C_25_H_28_N_2_O_5_: C, 68.79; H, 6.47; N, 6.42. Found: C, 69.03; H, 6.52; N, 6.43.

### Refinement

The hydrogen atom of the amino group was localized in the difference-Fourier map and refined isotropically with fixed isotropic displacement parameters [*U*_iso_(H) = 1.2*U*_eq_(N)]. The other hydrogen atoms were placed in calculated positions with C–H = 0.95–1.00 Å and refined in the riding model with fixed isotropic displacement parameters [*U*_iso_(H) = 1.5*U*_eq_(C) for the methyl group and 1.2*U*_eq_(C) for the other groups].

### Crystal data

**Table:**

C_25_H_28_N_2_O_5_	*F*(000) = 1856
*M**_r_* = 436.49	*D*_x_ = 1.334 Mg m^−^^3^
Orthorhombic, *P**b**c**a*	Mo *K*α radiation, λ = 0.71073 Å
Hall symbol: -P 2ac 2ab	Cell parameters from 6757 reflections
*a* = 17.1756 (6) Å	θ = 2.4–27.6°
*b* = 11.1724 (4) Å	µ = 0.09 mm^−^^1^
*c* = 22.6546 (8) Å	*T* = 100 K
*V* = 4347.3 (3) Å^3^	Prism, colourless
*Z* = 8	0.30 × 0.25 × 0.25 mm

### Data collection

**Table:**

Bruker APEXII CCD diffractometer	6326 independent reflections
Radiation source: fine-focus sealed tube	4682 reflections with *I* > 2σ(*I*)
Graphite monochromator	*R*_int_ = 0.069
φ and ω scans	θ_max_ = 30.0°, θ_min_ = 1.8°
Absorption correction: multi-scan (*SADABS*; Sheldrick, 2003)	*h* = −24→24
*T*_min_ = 0.973, *T*_max_ = 0.977	*k* = −15→15
54466 measured reflections	*l* = −31→31

### Refinement

**Table:**

Refinement on *F*^2^	Primary atom site location: structure-invariant direct methods
Least-squares matrix: full	Secondary atom site location: difference Fourier map
*R*[*F*^2^ > 2σ(*F*^2^)] = 0.042	Hydrogen site location: mixed
*wR*(*F*^2^) = 0.106	H atoms treated by a mixture of independent and constrained refinement
*S* = 1.00	*w* = 1/[σ^2^(*F*_o_^2^) + (0.0483*P*)^2^ + 1.18*P*] where *P* = (*F*_o_^2^ + 2*F*_c_^2^)/3
6326 reflections	(Δ/σ)_max_ < 0.001
293 parameters	Δρ_max_ = 0.34 e Å^−^^3^
0 restraints	Δρ_min_ = −0.24 e Å^−^^3^

### Special details

**Table:**

Geometry. All e.s.d.'s (except the e.s.d. in the dihedral angle between two l.s. planes) are estimated using the full covariance matrix. The cell e.s.d.'s are taken into account individually in the estimation of e.s.d.'s in distances, angles and torsion angles; correlations between e.s.d.'s in cell parameters are only used when they are defined by crystal symmetry. An approximate (isotropic) treatment of cell e.s.d.'s is used for estimating e.s.d.'s involving l.s. planes.
Refinement. Refinement of *F*^2^ against ALL reflections. The weighted *R*-factor *wR* and goodness of fit *S* are based on *F*^2^, conventional *R*-factors *R* are based on *F*, with *F* set to zero for negative *F*^2^. The threshold expression of *F*^2^ > σ(*F*^2^) is used only for calculating *R*-factors(gt) *etc*. and is not relevant to the choice of reflections for refinement. *R*-factors based on *F*^2^ are statistically about twice as large as those based on *F*, and *R*- factors based on ALL data will be even larger.

### Fractional atomic coordinates and isotropic or equivalent isotropic displacement parameters (Å^2^)

**Table:**

	*x*	*y*	*z*	*U*_iso_*/*U*_eq_	
C1	0.14643 (7)	0.45850 (10)	0.11628 (5)	0.0150 (2)	
H1	0.1927	0.4136	0.1012	0.018*	
C2	0.07641 (7)	0.40656 (10)	0.08499 (5)	0.0176 (2)	
C3	0.08519 (8)	0.30961 (11)	0.04720 (6)	0.0242 (3)	
H3	0.1359	0.2793	0.0398	0.029*	
C4	0.02150 (9)	0.25559 (12)	0.01993 (6)	0.0302 (3)	
H4	0.0288	0.1886	−0.0053	0.036*	
C5	−0.05235 (8)	0.30019 (12)	0.02980 (6)	0.0276 (3)	
H5	−0.0960	0.2627	0.0120	0.033*	
C6	−0.06308 (8)	0.39996 (12)	0.06580 (6)	0.0238 (3)	
H6	−0.1137	0.4321	0.0715	0.029*	
C7	0.00101 (7)	0.45237 (11)	0.09343 (5)	0.0194 (2)	
O8	−0.00310 (5)	0.54876 (8)	0.13029 (4)	0.0238 (2)	
C9	−0.07793 (7)	0.59780 (13)	0.14482 (6)	0.0262 (3)	
H9A	−0.1115	0.5361	0.1632	0.031*	
H9B	−0.1042	0.6285	0.1090	0.031*	
C10	−0.06217 (8)	0.69751 (13)	0.18737 (6)	0.0273 (3)	
H10A	−0.0258	0.7561	0.1696	0.033*	
H10B	−0.1112	0.7394	0.1973	0.033*	
O11	−0.02887 (5)	0.64651 (8)	0.23893 (4)	0.0245 (2)	
C12	0.01097 (7)	0.73098 (12)	0.27487 (6)	0.0244 (3)	
H12A	−0.0268	0.7808	0.2968	0.029*	
H12B	0.0435	0.7842	0.2501	0.029*	
C13	0.06106 (7)	0.66211 (12)	0.31701 (6)	0.0235 (3)	
H13A	0.0838	0.7167	0.3468	0.028*	
H13B	0.0296	0.6010	0.3378	0.028*	
O14	0.12189 (5)	0.60544 (8)	0.28352 (4)	0.01997 (18)	
C15	0.16485 (7)	0.51843 (11)	0.31095 (5)	0.0174 (2)	
C16	0.16080 (7)	0.49369 (12)	0.37114 (5)	0.0222 (3)	
H16	0.1278	0.5396	0.3960	0.027*	
C17	0.20549 (8)	0.40101 (12)	0.39451 (6)	0.0241 (3)	
H17	0.2017	0.3825	0.4353	0.029*	
C18	0.25533 (7)	0.33568 (12)	0.35910 (6)	0.0220 (3)	
H18	0.2856	0.2725	0.3753	0.026*	
C19	0.26073 (7)	0.36366 (11)	0.29901 (5)	0.0178 (2)	
H19	0.2962	0.3205	0.2749	0.021*	
C20	0.21530 (6)	0.45337 (10)	0.27385 (5)	0.0149 (2)	
C21	0.21819 (6)	0.47517 (10)	0.20788 (5)	0.0139 (2)	
H21	0.2596	0.4215	0.1917	0.017*	
C22	0.24208 (6)	0.60694 (10)	0.19154 (5)	0.0144 (2)	
H22	0.2505	0.6539	0.2286	0.017*	
C23	0.31604 (7)	0.61447 (11)	0.15252 (5)	0.0174 (2)	
H23A	0.3315	0.6993	0.1480	0.021*	
H23B	0.3593	0.5717	0.1723	0.021*	
N24	0.30278 (6)	0.56178 (9)	0.09399 (4)	0.0182 (2)	
C25	0.23402 (7)	0.60548 (11)	0.06232 (5)	0.0194 (2)	
H25A	0.2272	0.5590	0.0255	0.023*	
H25B	0.2417	0.6905	0.0515	0.023*	
C26	0.16073 (7)	0.59325 (10)	0.10078 (5)	0.0160 (2)	
H26	0.1145	0.6273	0.0798	0.019*	
N27	0.14364 (6)	0.43716 (9)	0.18055 (4)	0.01508 (19)	
H27	0.1049 (9)	0.4816 (13)	0.1957 (6)	0.018*	
C28	0.17742 (7)	0.66361 (10)	0.15626 (5)	0.0159 (2)	
O28	0.14686 (5)	0.75881 (8)	0.16807 (4)	0.02178 (19)	
C29	0.35168 (7)	0.48363 (12)	0.06601 (6)	0.0224 (3)	
O29	0.33441 (7)	0.43974 (10)	0.01789 (4)	0.0370 (3)	
C30	0.42915 (7)	0.45517 (12)	0.09395 (6)	0.0250 (3)	
H30A	0.4494	0.3803	0.0774	0.037*	
H30B	0.4225	0.4464	0.1367	0.037*	
H30C	0.4659	0.5203	0.0859	0.037*	

### Atomic displacement parameters (Å^2^)

**Table:**

	*U*^11^	*U*^22^	*U*^33^	*U*^12^	*U*^13^	*U*^23^
C1	0.0160 (5)	0.0145 (5)	0.0144 (5)	0.0005 (4)	−0.0013 (4)	−0.0007 (4)
C2	0.0214 (6)	0.0161 (5)	0.0153 (5)	−0.0028 (4)	−0.0044 (4)	0.0024 (4)
C3	0.0305 (7)	0.0199 (6)	0.0223 (6)	0.0040 (5)	−0.0115 (5)	−0.0018 (5)
C4	0.0432 (8)	0.0197 (6)	0.0276 (7)	0.0000 (6)	−0.0188 (6)	−0.0032 (5)
C5	0.0351 (7)	0.0251 (7)	0.0225 (6)	−0.0113 (6)	−0.0147 (6)	0.0067 (5)
C6	0.0200 (6)	0.0304 (7)	0.0212 (6)	−0.0071 (5)	−0.0041 (5)	0.0069 (5)
C7	0.0207 (6)	0.0207 (6)	0.0167 (5)	−0.0047 (5)	−0.0024 (4)	0.0033 (4)
O8	0.0147 (4)	0.0290 (5)	0.0278 (5)	−0.0003 (3)	0.0011 (3)	−0.0074 (4)
C9	0.0138 (5)	0.0349 (7)	0.0298 (7)	0.0033 (5)	0.0000 (5)	0.0000 (6)
C10	0.0203 (6)	0.0303 (7)	0.0312 (7)	0.0083 (5)	−0.0001 (5)	0.0006 (6)
O11	0.0229 (4)	0.0251 (5)	0.0254 (5)	0.0009 (4)	−0.0005 (4)	−0.0010 (4)
C12	0.0203 (6)	0.0234 (6)	0.0296 (7)	0.0037 (5)	0.0038 (5)	−0.0072 (5)
C13	0.0199 (6)	0.0281 (7)	0.0225 (6)	0.0034 (5)	0.0066 (5)	−0.0068 (5)
O14	0.0195 (4)	0.0211 (4)	0.0193 (4)	0.0053 (3)	0.0051 (3)	−0.0004 (3)
C15	0.0157 (5)	0.0191 (5)	0.0174 (5)	−0.0020 (4)	0.0000 (4)	−0.0015 (4)
C16	0.0217 (6)	0.0274 (6)	0.0173 (6)	−0.0020 (5)	0.0013 (5)	−0.0024 (5)
C17	0.0249 (6)	0.0326 (7)	0.0148 (5)	−0.0066 (5)	−0.0036 (5)	0.0025 (5)
C18	0.0214 (6)	0.0239 (6)	0.0206 (6)	−0.0034 (5)	−0.0072 (5)	0.0030 (5)
C19	0.0169 (5)	0.0180 (5)	0.0187 (5)	−0.0025 (4)	−0.0029 (4)	−0.0023 (4)
C20	0.0147 (5)	0.0153 (5)	0.0146 (5)	−0.0034 (4)	−0.0014 (4)	−0.0018 (4)
C21	0.0137 (5)	0.0138 (5)	0.0142 (5)	−0.0004 (4)	−0.0005 (4)	−0.0015 (4)
C22	0.0145 (5)	0.0132 (5)	0.0156 (5)	−0.0009 (4)	0.0018 (4)	−0.0028 (4)
C23	0.0153 (5)	0.0187 (5)	0.0182 (5)	−0.0020 (4)	0.0027 (4)	−0.0024 (4)
N24	0.0160 (5)	0.0218 (5)	0.0167 (5)	−0.0009 (4)	0.0027 (4)	−0.0018 (4)
C25	0.0184 (5)	0.0232 (6)	0.0166 (5)	−0.0019 (5)	0.0017 (4)	0.0027 (5)
C26	0.0162 (5)	0.0152 (5)	0.0165 (5)	−0.0007 (4)	0.0006 (4)	0.0024 (4)
N27	0.0154 (4)	0.0157 (5)	0.0141 (4)	−0.0021 (4)	−0.0012 (4)	−0.0002 (3)
C28	0.0143 (5)	0.0145 (5)	0.0190 (5)	−0.0026 (4)	0.0037 (4)	0.0019 (4)
O28	0.0217 (4)	0.0150 (4)	0.0286 (5)	0.0028 (3)	0.0010 (4)	−0.0012 (3)
C29	0.0234 (6)	0.0222 (6)	0.0215 (6)	0.0003 (5)	0.0039 (5)	−0.0020 (5)
O29	0.0422 (6)	0.0448 (6)	0.0238 (5)	0.0150 (5)	−0.0044 (4)	−0.0134 (4)
C30	0.0200 (6)	0.0283 (7)	0.0266 (6)	0.0009 (5)	0.0044 (5)	−0.0053 (5)

### Geometric parameters (Å, º)

**Table:**

C1—N27	1.4761 (14)	C16—C17	1.3936 (19)
C1—C2	1.5118 (16)	C16—H16	0.9500
C1—C26	1.5653 (16)	C17—C18	1.3816 (19)
C1—H1	1.0000	C17—H17	0.9500
C2—C3	1.3888 (17)	C18—C19	1.3999 (17)
C2—C7	1.4056 (17)	C18—H18	0.9500
C3—C4	1.3937 (18)	C19—C20	1.3923 (16)
C3—H3	0.9500	C19—H19	0.9500
C4—C5	1.381 (2)	C20—C21	1.5151 (15)
C4—H4	0.9500	C21—N27	1.4843 (14)
C5—C6	1.394 (2)	C21—C22	1.5725 (16)
C5—H5	0.9500	C21—H21	1.0000
C6—C7	1.3950 (17)	C22—C28	1.5076 (16)
C6—H6	0.9500	C22—C23	1.5497 (15)
C7—O8	1.3646 (15)	C22—H22	1.0000
O8—C9	1.4353 (15)	C23—N24	1.4686 (15)
C9—C10	1.498 (2)	C23—H23A	0.9900
C9—H9A	0.9900	C23—H23B	0.9900
C9—H9B	0.9900	N24—C29	1.3674 (16)
C10—O11	1.4199 (16)	N24—C25	1.4655 (15)
C10—H10A	0.9900	C25—C26	1.5370 (16)
C10—H10B	0.9900	C25—H25A	0.9900
O11—C12	1.4220 (16)	C25—H25B	0.9900
C12—C13	1.4978 (19)	C26—C28	1.5099 (16)
C12—H12A	0.9900	C26—H26	1.0000
C12—H12B	0.9900	N27—H27	0.898 (15)
C13—O14	1.4381 (14)	C28—O28	1.2159 (14)
C13—H13A	0.9900	C29—O29	1.2314 (16)
C13—H13B	0.9900	C29—C30	1.5074 (18)
O14—C15	1.3694 (14)	C30—H30A	0.9800
C15—C16	1.3930 (16)	C30—H30B	0.9800
C15—C20	1.4091 (16)	C30—H30C	0.9800
			
N27—C1—C2	112.01 (9)	C16—C17—H17	119.6
N27—C1—C26	112.44 (9)	C17—C18—C19	119.19 (12)
C2—C1—C26	112.88 (9)	C17—C18—H18	120.4
N27—C1—H1	106.3	C19—C18—H18	120.4
C2—C1—H1	106.3	C20—C19—C18	121.45 (11)
C26—C1—H1	106.3	C20—C19—H19	119.3
C3—C2—C7	117.89 (11)	C18—C19—H19	119.3
C3—C2—C1	120.13 (11)	C19—C20—C15	118.16 (10)
C7—C2—C1	121.96 (10)	C19—C20—C21	120.07 (10)
C2—C3—C4	121.72 (13)	C15—C20—C21	121.71 (10)
C2—C3—H3	119.1	N27—C21—C20	109.71 (9)
C4—C3—H3	119.1	N27—C21—C22	113.25 (9)
C5—C4—C3	119.53 (13)	C20—C21—C22	113.03 (9)
C5—C4—H4	120.2	N27—C21—H21	106.8
C3—C4—H4	120.2	C20—C21—H21	106.8
C4—C5—C6	120.32 (12)	C22—C21—H21	106.8
C4—C5—H5	119.8	C28—C22—C23	106.18 (9)
C6—C5—H5	119.8	C28—C22—C21	109.01 (9)
C5—C6—C7	119.60 (13)	C23—C22—C21	113.51 (9)
C5—C6—H6	120.2	C28—C22—H22	109.3
C7—C6—H6	120.2	C23—C22—H22	109.3
O8—C7—C6	124.40 (12)	C21—C22—H22	109.3
O8—C7—C2	114.73 (10)	N24—C23—C22	111.47 (9)
C6—C7—C2	120.87 (12)	N24—C23—H23A	109.3
C7—O8—C9	119.20 (10)	C22—C23—H23A	109.3
O8—C9—C10	105.64 (10)	N24—C23—H23B	109.3
O8—C9—H9A	110.6	C22—C23—H23B	109.3
C10—C9—H9A	110.6	H23A—C23—H23B	108.0
O8—C9—H9B	110.6	C29—N24—C25	118.73 (10)
C10—C9—H9B	110.6	C29—N24—C23	125.40 (10)
H9A—C9—H9B	108.7	C25—N24—C23	115.69 (9)
O11—C10—C9	107.69 (11)	N24—C25—C26	110.66 (9)
O11—C10—H10A	110.2	N24—C25—H25A	109.5
C9—C10—H10A	110.2	C26—C25—H25A	109.5
O11—C10—H10B	110.2	N24—C25—H25B	109.5
C9—C10—H10B	110.2	C26—C25—H25B	109.5
H10A—C10—H10B	108.5	H25A—C25—H25B	108.1
C10—O11—C12	113.49 (10)	C28—C26—C25	105.67 (9)
O11—C12—C13	107.48 (11)	C28—C26—C1	110.10 (9)
O11—C12—H12A	110.2	C25—C26—C1	109.95 (9)
C13—C12—H12A	110.2	C28—C26—H26	110.3
O11—C12—H12B	110.2	C25—C26—H26	110.3
C13—C12—H12B	110.2	C1—C26—H26	110.3
H12A—C12—H12B	108.5	C1—N27—C21	109.71 (9)
O14—C13—C12	107.90 (10)	C1—N27—H27	108.2 (9)
O14—C13—H13A	110.1	C21—N27—H27	108.8 (9)
C12—C13—H13A	110.1	O28—C28—C22	124.66 (11)
O14—C13—H13B	110.1	O28—C28—C26	123.82 (11)
C12—C13—H13B	110.1	C22—C28—C26	111.26 (9)
H13A—C13—H13B	108.4	O29—C29—N24	121.11 (12)
C15—O14—C13	117.69 (9)	O29—C29—C30	120.02 (12)
O14—C15—C16	123.92 (11)	N24—C29—C30	118.83 (11)
O14—C15—C20	115.27 (10)	C29—C30—H30A	109.5
C16—C15—C20	120.81 (11)	C29—C30—H30B	109.5
C15—C16—C17	119.46 (12)	H30A—C30—H30B	109.5
C15—C16—H16	120.3	C29—C30—H30C	109.5
C17—C16—H16	120.3	H30A—C30—H30C	109.5
C18—C17—C16	120.88 (12)	H30B—C30—H30C	109.5
C18—C17—H17	119.6		
			
N27—C1—C2—C3	−111.77 (12)	C19—C20—C21—N27	110.05 (11)
C26—C1—C2—C3	120.11 (12)	C15—C20—C21—N27	−67.13 (13)
N27—C1—C2—C7	67.29 (14)	C19—C20—C21—C22	−122.51 (11)
C26—C1—C2—C7	−60.83 (14)	C15—C20—C21—C22	60.31 (14)
C7—C2—C3—C4	−2.39 (19)	N27—C21—C22—C28	5.27 (12)
C1—C2—C3—C4	176.71 (12)	C20—C21—C22—C28	−120.29 (10)
C2—C3—C4—C5	1.0 (2)	N27—C21—C22—C23	−112.85 (10)
C3—C4—C5—C6	1.3 (2)	C20—C21—C22—C23	121.59 (10)
C4—C5—C6—C7	−2.06 (19)	C28—C22—C23—N24	−53.90 (12)
C5—C6—C7—O8	−178.82 (11)	C21—C22—C23—N24	65.84 (12)
C5—C6—C7—C2	0.61 (18)	C22—C23—N24—C29	−133.39 (12)
C3—C2—C7—O8	−178.95 (11)	C22—C23—N24—C25	51.51 (13)
C1—C2—C7—O8	1.97 (16)	C29—N24—C25—C26	131.12 (11)
C3—C2—C7—C6	1.57 (18)	C23—N24—C25—C26	−53.45 (13)
C1—C2—C7—C6	−177.51 (11)	N24—C25—C26—C28	57.62 (12)
C6—C7—O8—C9	3.31 (18)	N24—C25—C26—C1	−61.17 (12)
C2—C7—O8—C9	−176.15 (11)	N27—C1—C26—C28	6.70 (13)
C7—O8—C9—C10	177.69 (11)	C2—C1—C26—C28	134.60 (10)
O8—C9—C10—O11	−63.66 (13)	N27—C1—C26—C25	122.73 (10)
C9—C10—O11—C12	161.37 (10)	C2—C1—C26—C25	−109.37 (11)
C10—O11—C12—C13	−164.28 (10)	C2—C1—N27—C21	172.45 (9)
O11—C12—C13—O14	68.21 (13)	C26—C1—N27—C21	−59.19 (12)
C12—C13—O14—C15	−166.84 (10)	C20—C21—N27—C1	179.95 (9)
C13—O14—C15—C16	−9.56 (17)	C22—C21—N27—C1	52.63 (12)
C13—O14—C15—C20	170.68 (10)	C23—C22—C28—O28	−110.25 (12)
O14—C15—C16—C17	178.30 (11)	C21—C22—C28—O28	127.11 (12)
C20—C15—C16—C17	−1.95 (18)	C23—C22—C28—C26	64.03 (11)
C15—C16—C17—C18	1.83 (19)	C21—C22—C28—C26	−58.60 (11)
C16—C17—C18—C19	0.10 (19)	C25—C26—C28—O28	108.24 (12)
C17—C18—C19—C20	−1.97 (18)	C1—C26—C28—O28	−133.08 (11)
C18—C19—C20—C15	1.83 (17)	C25—C26—C28—C22	−66.11 (11)
C18—C19—C20—C21	−175.45 (10)	C1—C26—C28—C22	52.58 (12)
O14—C15—C20—C19	179.92 (10)	C25—N24—C29—O29	−8.16 (18)
C16—C15—C20—C19	0.15 (17)	C23—N24—C29—O29	176.88 (12)
O14—C15—C20—C21	−2.85 (16)	C25—N24—C29—C30	169.51 (11)
C16—C15—C20—C21	177.38 (11)	C23—N24—C29—C30	−5.45 (18)

### Hydrogen-bond geometry (Å, º)

**Table:**

*D*—H···*A*	*D*—H	H···*A*	*D*···*A*	*D*—H···*A*
N27—H27···O8	0.90 (2)	2.49 (2)	3.0337 (13)	119 (1)
N27—H27···O14	0.90 (2)	2.44 (1)	3.0193 (13)	122 (1)
C21—H21···O28^i^	1.00	2.48	3.4683 (14)	168
C30—H30*B*···O28^i^	0.98	2.51	3.0556 (16)	115

Symmetry code: (i) −*x*+1/2, *y*−1/2, *z*.
